# A Inibição do Metabolismo da Glicose por miR-34a e miR-125b Protege contra a Morte Celular de Cardiomiócitos Causada por Hiperglicemia

**DOI:** 10.36660/abc.20190529

**Published:** 2021-03-03

**Authors:** Chao-rui Xu, Qiu-ju Fang

**Affiliations:** 1 Heilongjiang Province Hospital Harbin China Heilongjiang Province Hospital, Harbin - China.; 2 Heilongjiang Province Hospital Department of Cardiology for the Elderly Harbin China Heilongjiang Province Hospital - Department of Cardiology for the Elderly, Harbin – China.

**Keywords:** Cardiomiopatia Diabética, Hiperglicemia, Transtornos Metabólicos de Glicose, Morte Celular, Miócitos Cardíacos, Ratos

## Abstract

**Fundamento:**

É sabido que a resistência à insulina e a hiperglicemia são causas patológicas importantes no desenvolvimento de cardiomiopatia diabética (CMD). Entretanto, seus mecanismos moleculares precisos na patogênese da CMD ainda não estão claros.

**Objetivos:**

Estudos recentes revelam que os microRNAs (miRNAs) desempenham papéis essenciais na patogênese da CMD. Este projeto tem o objetivo de determinar os papéis de miR-34a e miR-125b na morte celular de cardiomiócitos causada por hiperglicemia.

**Métodos:**

Cardiomiócitos primários de ratos foram isolados e expostos a concentrações de glicose normais e altas. A viabilidade das células foi medida utilizando-se o ensaio MTT. As expressões de miR-34a e miR-125b foram detectadas por qRT-PCR. Alvos potenciais de miR-34a e miR-125b foram previstos pelo www.Targetscan.org, e validados a partir de tecidos cardíacos humanos. Um p<0,05 foi considerado significância estatística.

**Resultados:**

Demonstra-se neste estudo que o miR-34a e o miR-125b têm resposta celular reduzida no coração humano diabético. Além disso, os dados *in vitro* de cardiomiócitos primários de ratos demonstraram que o tratamento com glicose alta em curto prazo estimula a expressão de miR-34a e miR-125b. Demonstrou-se que, em condições de glicose alta, os cardiomiócitos de ratos apresentaram metabolismo de glicose intracelular, e a captação de glicose e a produção de lactato aumentaram significativamente. Foi identificado que as principais enzimas metabólicas da glicose, hexoquinase 2 (HK2) e lactato desidrogenase-A (LDHA) eram alvos diretos de miR-125b e miR-34a, respectivamente. A superexpressão de miR-125b e miR-34a poderia evitar a morte de celular de cardiomiócitos causada por hiperglicemia. Por fim, a recuperação de HK2 e LDHA em cardiomiócitos com superexpressão de miR-125b e miR-34a restaurou a sensibilidade de cardiomiócitos à hiperglicemia.

**Conclusões:**

Nossos resultados propõem um mecanismo molecular para proteção cardiovascular diabética mediada por microRNA e contribuirão para o desenvolvimento de estratégias de tratamento de disfunção cardiovascular associada a diabetes.

## Introdução

A cardiomiopatia diabética (CMD), que está associada ao aumento da incidência de insuficiência cardíaca em pacientes diabéticos, é uma complicação cardíaca crônica e irreversível.^1^^,^^2^ Ela é caracterizada por alterações patopsicológicas complicadas na estrutura e na função do miocárdio, incluindo disfunção diastólica precoce, dilatação ventricular e hipertrofia cardíaca.^2^^,^^3^ A CMD é causada por fatores que são independentes de doença arterial coronariana (DAC), tais como resistência à insulina no tecido cardíaco, hiperinsulinemia compensatória e hiperglicemia.^4^ Atualmente, os mecanismos precisos que resultam em CMD ainda estão sendo investigados.

Altos níveis glicêmicos têm um papel importante em várias complicações diabéticas, incluindo a CMD, pela indução de reações inflamatórias.^5^ Após a captação pelas células, a glicose é quebrada em piruvato/lactato, um processo chamado glicólise anaeróbia.^6^ A glicólise é regulada em várias etapas reguladoras, tais como a captação da glicose, a fosforilação da glicose, e a conversão do piruvato em lactato ou Acetil-CoA.^7^ Estudos recentes relataram que a inibição de glicose alta por irisina pode afetar o desenvolvimento de CMD pela regulação da transição endotélio-mesenquimal (EndMT),^8^ o que sugere que o bloqueio da glicólise pode ser benéfico de pacientes de CMD. Além disso, outro estudo ilustrou que a metalotiona, um antioxidante, poderia inibir o stress oxidativo causado pela hiperglicemia, resultando na supressão da CMD.^9^ Os relatórios acima indicam que o bloqueio da hiperglicemia poderia evitar a CMD. Portanto, um entendimento melhor da patofisiologia das CMD será uma ajuda significativa para o diagnóstico precoce e o tratamento de disfunção cardiovascular associada a diabetes.

O MicroRNA, um pequeno (~20-25 nt) RNA altamente conservado e não codificante, já demonstrou ter papéis essenciais na remodelação cardíaca e no desenvolvimento de insuficiência cardíaca,^10^^,^^11^ sugerindo um alvo terapêutico potencial para o diagnóstico e o tratamento de CMD. Entre os microRNAs sobre os quais há relatos de alteração significativa durante as CMD, o miR-34a tende a aumentar durante a CMD,^11^ enquanto sabe-se que o miR-125b está associado ao crescimento hipertrófico,^12^ indicando que o miR-34a e o miR-125b estão envolvidos no desenvolvimento de CMD. Entretanto, ainda não está claro se o miR-125b e o miR-34a poderiam regular a cardiomiopatia causada por hiperglicemia. Portanto, serão investigados o possível papel e o mecanismo do miR-34a e do miR-125b na disfunção de cardiomiócitos causada por hiperglicemia, sugerindo uma nova estratégia terapêutica para lidar com a CMD.

## Métodos

### Cultura de cardiomiócitos de ratos

Foi realizado o isolamento de cardiomiócitos de ratos após o estudo prévio.^13^ Resumidamente, foram coletados cardiomiócitos de ratos neonatos com dois dias de vida. No total, 8 ratos foram dissecados, e todos os cardiomiócitos de ratos isolados/em cultura foram agrupados. Todos os experimentos foram realizados utilizando-se as mesmas células do grupo. Os cardiomiócitos foram ainda foram identificados por coloração com actina de músculo liso, alfa-actina sarcomérica e tropomiosina. Os cardiomiócitos foram colocados em cultura em meio de cardiomiócito específico (Nº de catálogo 6201; ScienCell), de acordo com as instruções do fabricante. O meio de cultura foi renovado a cada 24 horas. Após setenta e duas horas, o meio de cultura celular foi trocado pelo meio Eagle modificado por Dulbecco (DMEM, Nº de catálogo 31600-034; Invitrogen Corporation, Grand Island, NY, EUA) com 10% de soro fetal bovino (FBS) e 1% penicilina/estreptomicina em uma atmosfera umidificada com 5% CO_2_ a 37^o^C. Os experimentos com células de ratos foram realizados em triplicata e repetidos três vezes. Os experimentos com animais foram realizados com aprovação do Comitê Análise de Ética Animal do Hospital da Província de Heilongjiang (Nº AHPH-201711-06).

### Amostras de tecido cardíaco humano

Foram obtidas amostras de tecido cardíaco humano de corações humanos com insuficiência devido à CMD (20 casos) no momento do transplante no Hospital da Província de Heilongjiang. Os tecidos foram congelados imediatamente em nitrogênio líquido e, em seguida, armazenados a -80^o^C até o uso. Os tecidos de corações normais (20 casos) foram obtidos de doadores saudáveis, sem transplantes, e foram armazenados pelos mesmos procedimentos. A coleta de tecido humano foi realizada com a aprovação do Comitê Análise de Ética do Hospital da Província de Heilongjiang (Nº PH-201706-2H). O termo de consentimento informado foi obtido de todos os pacientes.

### Transfecção de precursores de microRNA e DNA plasmidial

O precursor de miR-34a, o precursor de miR-125b e o controle negativo foram comprados da Genepharma (Xangai, China). Os precursores de microRNA e o microRNA de controle negativo foram transfectados a 50 nM. As células foram semeadas em placas de 6 poços em densidades de 10^5^, 24 h antes da transfecção. A transfecção foi realizada utilizando-se o reagente de transfecção Lipofectamine RNAiMax (Invitrogen, Carlsbad, CA, EUA) de acordo com as instruções do fabricante. Após 72 horas, as células foram coletadas para análise a jusante. Vetores de superexpressão contendo ORF LDHA humana ou HK2 foram comprados da Origene Technologies Inc. (Rockville, MD), e foram transfectadas 4 µg de plasmídeos utilizando-se o reagente de transfecção Lipofectamine 2000 (Invitrogen, Carlsbad, CA, EUA) de acordo com o protocolo do fabricante. As células foram coletadas após 48 horas para análise a jusante.

### Captação de glicose e produção de lactato

O ensaio de captação de glicose foi realizado utilizando-se o kit de ensaio colorimétrico de captação de glicose (nº MAK083) da Sigma (Xangai, China), de acordo com as instruções do fabricante. A produção de lactato foi detectada utilizando-se o kit de ensaio de lactato (nº MAK064) da Sigma (Xangai, China), de acordo com as instruções do fabricante. Os dados foram normalizados pelo número de células de cada grupo experimental. Os ensaios foram realizados em triplicata e repetidos três vezes.

### Detecção de morte celular de cardiomiócitos

A morte celular de cardiomiócitos foi investigada pelo ensaio MTT (brometo de 3-(4,5-dimetiltiazol-2-yl)-2,5-difeniltetrazolio) e foi verificada por coloração azul de Trypan. Resumidamente, 24 horas após os tratamentos com glicose alta, o meio de cultura das células foi substituído por 200 *μ*l de meio fresco, e 20 *μ*L de MTT de 5 mg/mL (Sigma, nº M5655) foram colocados nos cardiomiócitos de ratos por 2 horas a 37 °C. O meio de cultura foi totalmente retirado, e foram adicionados 100 *μ*L de DMSO. As placas foram colocadas no agitador orbital por 5 minutos em temperatura ambiente. A absorbância na densidade óptica (OD) de 590 nM foi medida. A absorbância foi normalizada pelo número de células de cada poço. Cada experimento foi realizado em triplicata e repetido três vezes.

### RT-PCR quantitativo

O RNA total foi isolado de células de cardiomiócitos e tecido utilizando-se o reagente TRIzol (Invitrogen, Carlsbad, CA) de acordo com as instruções do fabricante. Depois do tratamento com DNase, a qualidade do RNA foi medida pelo espectrofotômetro NanoDrop. Para detecção dos miRNAs, foi conjugada uma cauda poliA ao RNA digerido por DNase livre de RNase, e o qRT-PCR foi realizado utilizando-se o kit de detecção de miRNA (Applied Biosystems) de acordo com as instruções do fabricante. O gene U6 humano foi utilizado como controle interno. A reação em cadeia da polimerase quantitativa (qPCR) foi realizada utilizando-se SYBR Green Taq ReadyMix (Sigma) em um sistema de PCR Applied Biosystems7500. Os resultados foram analisados pelo método 2^−ΔΔCt^ e normalizados pela expressão do gene U6. Todos os ensaios foram realizados com o sistema de detecção de PCR em tempo real multicolor Bio-Rad IQTM5. Os experimentos foram realizados em triplicata e repetidos três vezes.

### Previsão de alvo

Os alvos potenciais de miR-125b e miR-34a foram previstos por computação pelo programa TargetScan (http://www.targetscan.org/).

### Análise Western Blot

A análise Western Blot foi realizada para avaliar as expressões das proteínas HK2 e LDHA. Lisados celulares de cardiomiócitos de ratos foram extraídos utilizando-se o tampão de lise e extração RIPA (#89900, Thermo Scientific, Xangai, China). A concentração de proteína foi determinada pelo método de Bradford conforme previamente descrito.^14^ Uma quantidade igual de amostra de proteína foi adicionada ao gel SDS-PAGE a 10%, seguida de eletroforese, e transferida para membrana de nitrocelulose. As membranas foram bloqueadas por BSA a 5% por uma hora em temperatura ambiente. Depois de uma lavagem completa com TBST, as manchas foram incubadas com os anticorpos primários da Cellsignaling Technology (Danvers, MA, EUA) (HK2, #2867; LDHA, #3582 e α-tubulina, #2125) a 1:1000, durante toda a noite, a 4 oC. As membranas foram lavadas e incubadas com os anticorpos secundários respectivos a 1:3000 por uma hora em temperatura ambiente. As bandas foram detectadas por agentes desenvolvedores de quimioluminescência (SuperSignal, Thermo Scientific). Os resultados foram repetidos três vezes e números representativos foram apresentados.

### Análise estatística

A análise estatística foi realizada com o software GraphPad Prism 5 (GraphPad Software, Inc., La Jolla, CA, EUA). O teste t de Student não pareado foi utilizado para análise de dados entre dois grupos. A significância entre três ou mais grupos foi analisada pelo ANOVA. Os dados foram apresentados como média e desvio padrão. As barras de erro nos gráficos representam o desvio padrão. As correlações entre as variáveis foram determinadas pelo coeficiente de correlação de Pearson. Os experimentos foram realizados em triplicata e repetidos três vezes. Os dados dos experimentos com cardiomiócitos de rato foram normalizados pelo número de células de cada grupo experimental. A α-tubulina foi o controle interno para o teste Western blot. O gene U6 foi o controle interno do qRT-PCR. Um *p* <0,05 foi considerado estatisticamente significativo.

## Resultados

### Resposta celular reduzida em miR-34a e miR-125b no coração humano diabético

**Figura 1:**
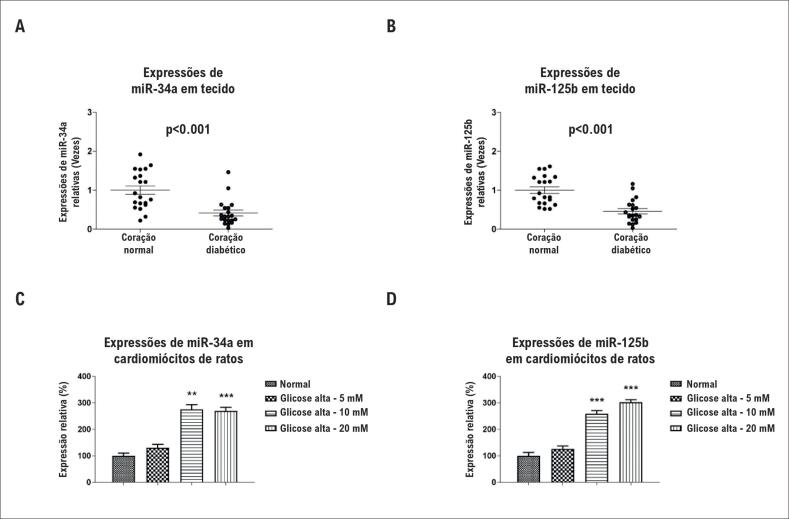
Resposta celular reduzida em miR-34a e miR-125b no coração humano diabético induzida por hiperglicemia. (A) Expressões de miR-34a e (B) miR-125b no coração humano normal e em tecidos cardíacos diabéticos. (C) Cardiomiócitos de ratos foram tratados com glicose alta ou de controle em 5, 10 ou 20 mM. As expressões relativas do miR-34a e (D) do miR-125b foram avaliadas por PCR em tempo real e normalizadas aos níveis de snRNA U6. As colunas são a média de três experimentos independentes; as barras de erro nos gráficos representam o DP. **, p < 0,01; ***, p < 0,001.

Para avaliar os papéis dos miRNAs no coração humano diabético, foram examinados os níveis de expressão do miR-34a e do miR-125b em tecidos cardíacos humanos obtidos de 20 pacientes com insuficiência cardíaca diabética e 20 doadores saudáveis. As expressões do miR-34a e do miR-125b tiveram resposta celular significativamente reduzida nos tecidos cardíacos com insuficiência cardíaca diabética em comparação com os de doadores saudáveis (Figura 1A e 1B). Esses resultados sugerem uma função protetiva do miR-34a e do miR-125b na insuficiência cardíaca mediada por glicose alta.

### A glicose alta de curto prazo estimula as expressões de miR-34a e miR-125b em cardiomiócitos de ratos neonatos

Para avaliar o efeito do miR-34a e do miR-125b na disfunção de cardiomiócitos causada por glicose alta, foi estabelecido um modelo *in vitro* utilizando cardiomiócitos de rato isolados em cultura em condições normal e de glicose alta. É interessante notar que, em curto prazo (1 hora), detectou-se que o miR-34a e miR-125b foram significativamente induzidos por uma concentração de glicose alta de 25 ou 50 mM (Figura 1C e 1D). Entretanto, em condições de hiperglicemia de longo prazo (48 horas, 50 mM), os cardiomiócitos sofreram morte celular (dados não mostrados). Tomados em conjunto, os resultados acima demonstraram um aumento de miR-34a e miR-125b por hiperglicemia em cardiomiócitos.

### A superexpressão de miR-34a e miR-125b protege contra a morte celular de cardiomiócitos causada por hiperglicemia

**Figura 2:**
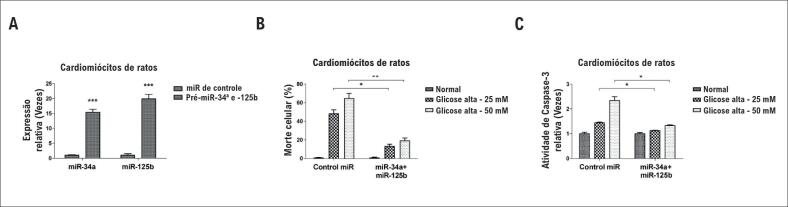
A superexpressão de miR-34a e miR-125b protege contra morte celular de cardiomiócitos causada por hiperglicemia (A) Cardiomiócitos primários de ratos foram transfectados com microRNA de controle, ou pré-miR-34a mais miR-125b por 72 horas. As expressões de miR-34a e miR-125b foram medidas por qRT-PCR. (B) Cardiomiócitos primários de ratos foram transfectados com microRNA de controle, ou pré-miR-34a mais miR-125b por 72 horas, as células foram expostas em condições de glicose normal ou alta (25 mM ou 50 mM) por 48 horas. O índice de apoptose celular foi medido pelo ensaio de MTT e (C) ensaio de atividade de Caspase-3. As colunas são a média de três experimentos independentes; as barras de erro nos gráficos representam o DP. *, p < 0,05; **, p < 0,01; ***, p < 0,001.

Sabia-se que a hiperglicemia poderia desencadear uma reação inflamatório e causar a morte celular de cardiomiócitos (4). Dessa forma, foi considerada a hipótese de a superexpressão exógena miR-34a e miR-125b poder proteger contra a morte celular de cardiomiócitos causada por hiperglicemia. Os cardiomiócitos de ratos foram cotransfectados com pré-miR-34a e pré-miR-125b por 48 horas. Os resultados do qRT-PCR demonstraram que as expressões de miR-34a e miR-125b foram aumentadas em até 5-10 vezes (Figura 2A). Em seguida, as células foram expostas a 25 ou 50 mM de glicose para simular a hiperglicemia por 48 horas. Como esperado, cardiomiócitos transfectados de miRNAs de controle apresentaram, obviamente, morte celular com o tratamento por hiperglicemia (Figura 2B). Entretanto, cardiomiócitos com superexpressão de miR-34a e miR-125b mostraram-se significativamente resistentes à hiperglicemia, tanto pelo ensaio MTT e pelo ensaio de atividade de Caspase-3 (Figura 2B e 2C). Esses resultados demonstraram um papel protetivo do miR-34a e do miR-125b na morte celular de cardiomiócitos causada por hiperglicemia.

### Inibição da glicólise pelo miR-34a e o miR-125b em hiperglicemia ao atingir LDHA e HK2

**Figura 3:**
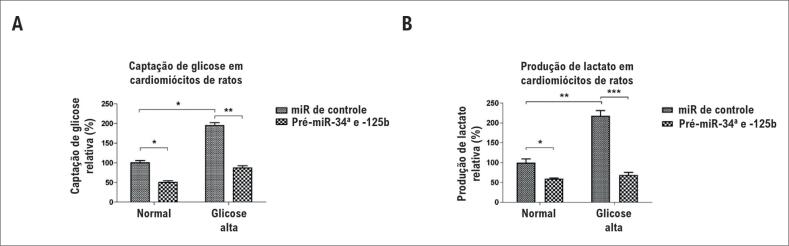
A superexpressão de miR-34a e miR-125b afeta o metabolismo da glicose induzido por hiperglicemia. (A) Cardiomiócitos primários de ratos foram transfectados com microRNA de controle, ou pré-miR-34a mais miR-125b por 72 horas, as células foram expostas em condições de glicose normal ou alta (25 mM) por 48 horas. A captação de glicose e (B) a produção de lactato foram medidas. As colunas são a média de três experimentos independentes; as barras de erro nos gráficos representam o DP. *, p < 0,05; **, p < 0,01; ***, p < 0,001.

**Figura 4:**
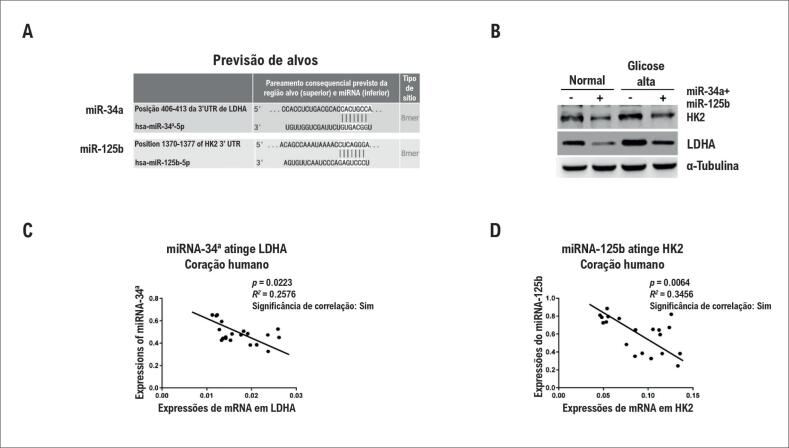
O miR-34a e o miR-125b atingem enzimas da glicólise em cardiomiócitos e tecidos cardíacos. (A) ilustração da LDHA 3’UTR e da HK2 3’UTR, bem como da sequência de semeadura de miR-34a e miR-125b, mostrando a região alvo prevista por computação na 3’UTR dos mRNAs da LDHA e HK2. (B) Cardiomiócitos de ratos foram transfectados com 25 nM de precursores de miR-34a e miR-125b por 72 horas. A superexpressão do miR-34a e do miR-125b teve resposta celular reduzida de expressões proteicas de LDHA e HK2 condições de glicose normal e alta. A α-tubulina foi um controle de carregamento. (C) Correlação negativa entre miR-34a e expressões de mRNA de LDHA em tecidos cardíacos humanos normais. (D) Correlação negativa entre miR-125b e expressões de mRNA de HK2 em tecidos cardíacos humanos normais.

Em condições de glicose alta, as células apresentaram um aumento no metabolismo da glicose.^6^^,^^7^ Para investigar os mecanismos subjacentes aos papéis do miR-34a e do miR-125b durante o tratamento de hiperglicemia, foi feita a revisão da literatura e foram encontrados estudos recentes que mostram que tanto o miR-34a quanto o miR-125b poderiam inibir o metabolismo da glicose intracelular.^15^^,^^16^ Portanto, levantou-se a hipótese de que a inibição da glicólise intracelular ativada por hiperglicemia pelo miR-34a e pelo miR-125b contribui para a proteção dos cardiomiócitos contra a morte celular. Para avaliar os efeitos inibitórios do miR-34a e do miR-125b na glicólise em hiperglicemia, foram medidas a captação de glicose e a produção de lactato de cardiomiócitos com ou sem superexpressão de miRNAs em tratamento com glicose normal ou alta. A superexpressão do miR-34a e do miR-125b inibiu a captação de glicose e a produção de lactato em glicose normal (Figura 3A e 3B). Além disso, os cardiomiócitos com miR-34a e miR-125b altos também apresentaram glicólise altamente diminuída, próxima aos níveis normais (Figura 3A e 3B), sugerindo que a superexpressão de miR-34a e miR-125b em condições de hiperglicemia contribui para a manutenção da homeostase da glicose intracelular. Para verificar os alvos diretos do miR-34a e do miR-125b nos cardiomiócitos, foram detectadas as expressões proteicas de LDHA, que é um alvo previsto do miR-34a, e de HK2, cuja 3’UTR poderia ser atingida diretamente pelo miR-125b (Figura 4A). Os resultados do teste de Western Blot demonstraram consistentemente que os níveis de HK2 e LDHA foram suprimidos pela superexpressão de miR-34a e miR-125b em condições de glicose normal ou hiperglicemia (Figura 4B). Foram verificados, além disso, os alvos do miR-34a e do miR-125b em tecido cardíaco humano. De maneira consistente, em miR-34a e miR-125b, cuja expressão é relativamente alta em tecidos cardíacos normais, os níveis de HK2 e LDHA de mRNA foram aparentemente baixos (Figura 4C). A mesma correlação negativa entre miR-34a e LDHA, e entre miR-125b e HK2 foi observada em tecidos cardíacos diabéticos (Figura 4D). Em geral, os dados confirmam que o miR-34a e o miR-125b inibem a glicólise intracelular atingindo enzimas que limitam a velocidade da glicólise, contribuindo para a manutenção da homeostase da glicose em hiperglicemia.

### A restauração de LDHA e HK2 sensibiliza os cardiomiócitos à glicose alta

**Figura 5:**
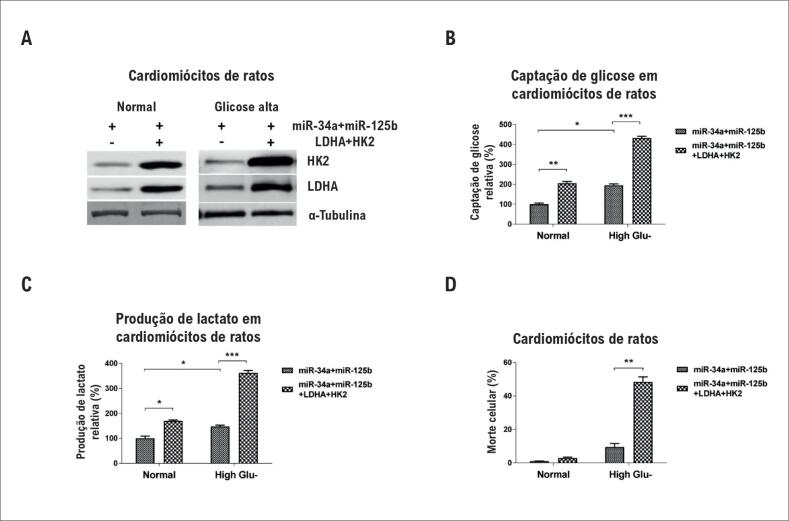
A restauração de glicólise em cardiomiócitos de ratos com superexpressão de miR-34a e miR-125b promove a morte celular em hiperglicemia. (A) Os cardiomiócitos de ratos foram transfectados com mistura de miR-34a e miR-125b sozinhos ou cotransfectados com miR-34a e plasmídeos de superexpressão de miR-125b e plasmídeos de superexpressão de LDHA e HK2 por 72 horas. As células foram tratadas com ou sem glicose alta (25 mM) por 48 horas e submetidas à a análise de Western Blot. A α-tubulina foi um controle de carregamento. (B) Os cardiomiócitos de ratos foram transfectados com mistura de miR-34a e miR-125b sozinhos ou cotransfectados com miR-34a e plasmídeos de superexpressão de miR-125b e plasmídeos de superexpressão de LDHA e HK2 por 72 horas. As células foram tratadas com ou sem glicose alta (25 mM) por 48 horas. A captação de glicose e (C) a produção de lactato foram medidas. (D) Os cardiomiócitos de ratos foram transfectados com mistura de miR-34a e miR-125b sozinhos ou cotransfectados com miR-34a e plasmídeos de superexpressão de miR-125b e plasmídeos de superexpressão de LDHA e HK2 por 72 horas. As células foram tratadas com ou sem glicose alta (50 mM) por 48 horas. A morte celular foi avaliada pelo ensaio de MTT. As colunas são a média de três experimentos independentes; as barras de erro nos gráficos representam o DP. *, p < 0,05; **, p < 0,01; ***, p < 0,001.

Por fim, para testar se a proteção de cardiomiócitos em hiperglicemia foi causada diretamente pela inibição da glicólise por miR-34a e miR-125b, foram realizados experimentos de resgate por cotransfecção de plasmídeos de superexpressão de LDHA e HK2 em cardiomiócitos de superexpressão de miR-34a e miR-125b. Os resultados do teste de Western Blot (Figura 5A) demonstraram que a cotransfecção de plasmídeos conseguiu resgatar com sucesso as expressões de LDHA e HK2 em cardiomiócitos de superexpressão de miR-34a e miR-125b. Além disso, a captação de glicose (Figura 5B) e a produção de lactato (Figura 5C) também foram trazidas a níveis normais pela restauração de LDHA e HK2. As células transfectadas acima foram expostas a 50 mM de glicose para simular a hiperglicemia por 48 horas. As células foram coletadas e submetidas a teste de morte celular. Como esperado, os cardiomiócitos com resgate de LDHA e HK2 apresentaram aumento de morte celular em hiperglicemia, em comparação com os índices dos cardiomiócitos com superexpressão de miR-34a e miR-125b (Figura 5D). Esses experimentos de resgate confirmaram a homeostase da glicose intracelular mediada por miR-34a e miR-125b em cardiomiócitos protegidos diretamente por hiperglicemia.

## Discussão

A CMD é uma das principais ameaças à saúde de pacientes com diabetes.^1^^,^^2^ Ela está associada a eventos patofisiológicos complexos, incluindo inflamação crônica, morte de células cardíacas, que podem levar à insuficiência cardíaca. A respostas cardíaca inicial ao diabetes foi a morte de cardiomiócitos apoptóticos.^3^ Neste estudo, relatou-se um mecanismo de proteção de cardiomiócitos mediados por microRNA em condições de hiperglicemia. Cardiomiócitos de ratos foram tratados com glicose alta e detectou-se aumento significativo de glicólise, um processo regulado pelo aumento adaptativo de miR-34a e miR-125b, indicando que atingir o miR-34a e o miR-125b no metabolismo da glicose causado por hiperglicemia pode contribuir para o desenvolvimento de um método terapêutico de proteção contra a morte de células cardíacas.

Glicose e a resistência aguda a insulina foram detectadas em problemas cardíacos agudos.^4^ Além disso, o metabolismo de glicose intracelular alta foi reconhecido como um marcador prognóstico potencial em síndromes coronárias agudas.^17^ A associação entre hiperglicemia e CMD foi estudada exaustivamente.^18^ A sinalização metabólica de insulina anormal, a hiperglicemia, e disfunção mitocondrial e o stress oxidativo são os mecanismos patofisiológicos envolvidos no desenvolvimento da CMD mais notórios.^18^ Atualmente, os mecanismos moleculares subjacentes que resultam em CMD não são suficientemente entendidos. Estudos recentes indicam que os microRNAs desempenham papéis essenciais na etiologia do diabetes e suas complicações.^11^ Além disso, relatou-se que o miR-125b e o miR-34a estão associados ao stress oxidativo de tecidos cardíacos, levando à inibição de morte celular de cardiomiócitos.^11^ Em cânceres, já foi relatado que o miR-34a e o miR-125b diminuíram e inibiram o metabolismo da glicose,^15^^,^^19^ o que sugere que o miR-34a e o miR-125b poderiam regular as células disfuncionais em condições de hiperglicemia. Os resultados demonstraram que miR-34a e miR-125b tiveram resposta celular significativamente reduzida no em tecidos cardíacos humanos diabéticos, em comparação com normais. Além disso, detectou-se que o miR-34a e o miR-125b eram estimulados de forma adaptativa em cardiomiócitos de ratos em condições de glicose alta, sugerindo que o miR-34a e o miR-125b pode suprimir o metabolismo da glicose intracelular causado por hiperglicemia. Já se sabia que o miR-34a poderia atingir a 3’UTR de mRNA de LDHA^20^ e que a HK2 era um alvo direto do miR-125b em células cancerígenas.^19^ Este estudo tem algumas limitações na confirmação dos efeitos dos miRNAS na proteção de CMD de dados *in vivo*. Os dados, entretanto, revelaram, pela primeira vez, que o miR-34a e a HK2 poderiam suprimir a captação de glicose e a produção de lactato em cardiomiócitos. A superexpressão do miR-34a e do miR-125b contribuiu para a manutenção do metabolismo da glicose intracelular em condições de hiperglicemia (Figura 3).

## Conclusão

Em resumo, este estudo demonstrou que o miR-34a e o miR-125b estão significativamente correlacionados à CMD humana. Utilizando um modelo de cardiomiócitos de ratos *in vitro*, a hiperglicemia estimula, de forma adaptativa, as expressões do miR-34a e do miR-125b. A superexpressão de miR-34a e de miR-125b suprimiu o metabolismo da glicose intracelular induzida por hiperglicemia atingindo LDHA e HK2, o que resultou na inibição da morte celular de cardiomiócitos induzida por hiperglicemia. Conjuntamente, os dados revelaram os possíveis papéis desempenhados pelo miR-34a e pelo miR-125b na patogênese da cardiomiopatia induzida por hiperglicemia. Nosso trabalho futuro focará em um modelo de rato diabético *in vivo* para investigar os mecanismos moleculares do miR-34a e do miR-125b na CMD.
