# Supplementary material for: A Inibição do Metabolismo da Glicose por miR-34a e miR-125b Protege contra a Morte Celular de Cardiomiócitos Causada por Hiperglicemia
Source: Arq Bras Cardiol. 2021 Mar 3;116(3):415–22. [Article in Portuguese] doi: 10.36660/abc.20190529 (PMC8159564; doi:10.36660/abc.20190529)
Supplement: Translation

# Translation: Inhibiting Glucose Metabolism By miR-34a and miR-125b Protects Against Hyperglycemia-Induced Cardiomyocyte Cell Death

**Authors:** Chao-rui Xu, Qiu-ju Fang

**Affiliations:** 1 Harbin China Heilongjiang Province Hospital, Harbin - China.; 2 Harbin China Heilongjiang Province Hospital - Department of Cardiology for the Elderly, Harbin – China.

**Keywords:** Hiperglycemia, Glicose Metabolism Disordes, Cell Death, Diabetic Cardiomyopathies, Myocytes,Cardiac, Rats

## Abstract

**Background:**

It is well-known that insulin resistance and hyperglycemia are important pathological causes for the development of diabetic cardiomyopathy (DCM). However, its precise molecular mechanisms in the pathogenesis of DCM remain unclear.

**Objectives:**

Recent studies reveal that microRNAs (miRNA) play essential roles in the pathogenesis of DCM. This project aimed to determine the roles of miR-34a and miR-125b in hyperglycemia-induced cardiomyocyte cell death.

**Methods:**

Rat primary cardiomyocytes were isolated and exposed to normal and high concentrations of glucose. Cell viability was measured using MTT assay. Expressions of miR-34a and miR-125b were detected by qRT-PCR. Potential targets of miR-34a and miR-125b were predicted from www.Targetscan.org and validated from human heart tissues. A statistical significance of p<0.05 was considered.

**Results:**

The present study shows that miR-34a and miR-125b are downregulated in a human diabetic heart. Moreover, in vitro data from rat primary cardiomyocytes showed that short-term high glucose treatment stimulates miR-34a and miR-125b expressions. Under high glucose, it was found that rat cardiomyocytes displayed increased intracellular glucose metabolism, and glucose uptake and lactate production were significantly increased. It was also found that the key glucose metabolic enzymes, Hexokinase 2 (HK2) and Lactate dehydrogenase-A (LDHA), were direct targets of miR-125b and miR-34a, respectively. Overexpression of miR-125b and miR-34a could prevent hyperglycemia-induced cardiomyocyte cell death. Finally, the restoration of HK2 and LDHA in miR-125b and miR-34a overexpressed cardiomyocytes recovered the cardiomyocytes’ sensitivity to hyperglycemia.

**Conclusion:**

Our results proposed a molecular mechanism for the microRNA-mediated diabetic cardiovascular protection and will contribute to developing treatment strategies for diabetes-associated cardiovascular dysfunction.

## Introduction

Diabetic cardiomyopathy (DCM), which is associated with an increased incidence of heart failure in diabetic patients, is a chronic and irreversible heart complication.^1^^,^^2^ It is characterized by complicated pathophysiologic changes in the structure and function of the myocardium, including early diastolic dysfunction, ventricular dilation, and cardiac hypertrophy.^2^^,^^3^ DCM is promoted by factors which are independent from coronary artery disease (CAD), such as insulin resistance in heart tissue, compensatory hyperinsulinemia, and hyperglycemia.^4^ Currently, the precise mechanisms resulting in DCM are still under investigation.

High glucose plays an important role in several diabetic complications including DCM through the induction of inflammatory reactions.^5^ Following uptake by cells, glucose is broken down into pyruvate/lactate, a process called anerobic glycolysis.^6^ Glycolysis is regulated at several rate-limiting steps, such as glucose uptake, glucose phosphorylation, and conversion of pyruvate into lactate or Acetyl-CoA.^7^ Recent studies reported high glucose inhibition by irisin could influence the development of DCM by regulating the endothelial to mesenchymal transition (EndMT),^8^ suggesting tha tblocking glycolysis may benefit DCM patients. In addition, another study illustrated metallothione, an antioxidant, could inhibit the hyperglycemia-induced oxidative stress, resulting in the suppression of DCM.^9^ The above reports indicate blocking the hyperglycemia could prevent the DCM. Thus, a better understanding of DCM’s pathophysiology will greatly benefit early diagnosis and the treatment for diabetes-associated cardiovascular dysfunction.

MicroRNA, a small (~20-25 nt) and highly conserved non-coding RNA, has proved to play critical roles in cardiac remodeling and the development of heart failure,^10^^,^^11^ suggesting a potentially therapeutic target for the diagnosis and treatment of DCM. Among microRNAs, which have been reported to be significantly altered during DCM, miR-34a tends be upregulated during DCM,^11^ while miR- 125b is known to be associated with hypertrophic growth,^12^ indicating that miR-34a and miR-125 are involved in the development of DCM. However, whether miR-125b and miR-34a could regulate the hyperglycemia-induced cardiomyopathy remains unclear. Therefore, the present study seeks to investigate the potential role and mechanism of miR-34a and miR-125b in hyperglycemia-induced cardiomyocyte dysfunction, suggesting a new therapeutic strategy in the management of DCM.

## Methods

### Rat cardiomyocyte culture

The isolation of rat cardiomyocytes was performed following prior study.^13^ Briefly, rat cardiomyocytes were collected from day two postnatal rat hearts. In total, eight rats were dissected, and the isolated/cultured cardiomyocytes from all rat hearts were pooled. All experiments were performed using the same cells from the pool. The cardiomyocytes were further identified by staining the smooth muscle actin, sarcomeric alpha-actinin, and tropomyosin. The cardiomyocytes were cultured with specific cardiomyocyte medium (Catalog No. 6201; ScienCell), according to manufacturer’s instructions. The culture medium was refreshed every 24 hours. Seventy-two hours later, cell culture medium was changed to serum-free Dulbecco’s modified Eagle’s medium (DMEM, Catalog No. 31600-034; Invitrogen Corporation, Grand Island, NY, USA), together with 10% Fetal Bovine Serum (FBS) and 1% penicillin/streptomycin in a humidified atmosphere of 5% CO_2_ at 37°C. Rat cell experiments were performed in triplicate and repeated three times. The animal experiments were performed after having received approval from the Animal Ethical Review Board of Heilongjiang Province Hospital (No. AHPH-201711-06).

### Human heart tissue samples

Human heart tissue samples were obtained from failing human hearts with DCM (20 cases) at the time of transplant at the Heilongjiang Province Hospital. Tissues were frozen immediately in liquid nitrogen and were then stored at -80°C until use. Normal hearts (20 cases) were obtained from healthy donors without transplants and stored by the same procedures. Human tissue collection was performed after having received approval from the Ethical Review Board of Heilongjiang Province Hospital (No. PH-201706-2H). Informed consent was obtained from all patients.

### Plasmid DNA and microRNA precursor transfection

MiR-34a precursor, miR-125b precursor, and negative control were purchased from Genepharma (Shanghai, China). MicroRNA precursors and negative control microRNA were transfected at 50 nM. Cells were seeded in 6-well plates at 10^5^ densities 24 h prior to transfection. Transfection was performed using the Lipofectamine RNAiMax Transfection Reagent (Invitrogen, Carlsbad, CA, USA) according to the manufacturer’s instructions. After 72 h, the cells were collected for downstream analysis. Overexpression vectors containing human ORF LDHA, or HK2, were purchased from Origene Technologies Inc. (Rockville, MD) and 4 ug of plasmid was transfected using the Lipofectamine 2000 transfection reagent (Invitrogen, Carlsbad, CA, USA) according to the manufacturer’s protocol. Cells were collected after 48 hours for downstream analysis.

### Glucose uptake and lactate production

The glucose uptake assay was performed using the Glucose Uptake Colorimetric Assay Kit (#MAK083) from Sigma (Shanghai, China), according to manufacturer’s instructions. The lactate production was detected using the Lactate Assay Kit (#MAK064) from Sigma (Shanghai, China), according to manufacturer’s instructions. Data were normalized by the cell numbers of each experimental group. Assays were performed in triplicate and repeated three times.

### Detection of cardiomyocyte cell death

The cardiomyocyte cell death was investigated by the MTT (3-(4,5-dimethylthiazol-2-yl)-2,5-diphenyltetrazolium bromide) assay and verified by direct Trypan blue staining. Briefly, 24 hours after treatments with high glucose, the cell culture medium was replaced with 200 *μ*l fresh medium and 20 *μ*L of 5 mg/mL MTT (Sigma, #M5655) was put into the rat cardiomyocytes for 2 hours at 37°C. The culture medium was completely removed, and 100 µL of DMSO was added. Plates were put on an orbital shaker for 5 minutes at room temperature. The absorbance, at optical density (OD) of 590 nm, was measured. This absorbance was normalized by cell numbers of each well. Each experiment was performed in triplicate and repeated three times.

### Quantitative RT-PCR

Total RNA was isolated from tissue and cardiomyocyte cells, using the TRIzol Reagent (Invitrogen, Carlsbad, CA) according to manufacturer’s instructions. After DNase treatment, the quality of the RNA was measured by NanoDrop. To detect the miRNAs, a polyA tail was conjugated to the RNase-free DNase digested RNA, and qRT-PCR was performed using the qRT-PCR miRNA Detection Kit (Applied Biosystems), following manufacturer’s instructions. Human U6 served as an internal control. Quantitative polymerase chain reaction (qPCR) was performed using SYBR Green Taq ReadyMix (Sigma) on an Applied Biosystems7500 PCR system. Results were analyzed using the 2^−ΔΔCt^ method and normalized to U6 expression. All qRT-PCR assays were performed with the Bio-Rad IQTM5 Multicolour Real-Time PCR Detection System. Experiments were performed in triplicate and repeated three times.

### Target prediction

The potential targets of miR-125b and miR-34a were computationally predicted by the TargetScan program (http://www.targetscan.org/).

### Western blot analysis

Western blot analysis was performed to assess the expressions of HK2 and LDHA proteins. Cell lysates from rat cardiomyocytes were extracted using RIPA Lysis and Extraction Buffer (#89900, Thermo Scientific, Shanghai, China). Protein concentration was determined by applying the Bradford method, as previously described.^14^ An equal amount of protein sample was loaded on a 10% SDS-PAGE gel, followed by electrophoresis, and transferred onto a nitrocellulose membrane. Membranes were blocked by 5% BSA for one hour at room temperature. After complete washing by TBST, the blots were incubated with primary antibodies, all from Cellsignaling Technology (Danvers, MA, USA) (HK2, #2867; LDHA, #3582 and α-tubulin, #2125) at 1:1000 for overnight at 4°C. Membranes were washed and incubated with respective secondary antibodies at 1:3000 for 1 hour at room temperature. Bands were detected by chemiluminescence developing agents (SuperSignal, Thermo Scientific). Results were repeated three times, and representative figures were shown.

### Statistical analysis

Statistical analysis was performed using the GraphPad Prism 5 software (GraphPad Software, Inc., La Jolla, CA, USA). The unpaired Student’s t-test was used for data analysis between two groups. The significance among three or more groups was analyzed by ANOVA. Data was presented as mean and standard deviation. The error bars in graphs represented standard deviation. Correlations between variables were determined by Pearson’s correlation coefficient. Experiments were performed in triplicate and repeated three times. Data from rat cardiomyocyte experiments were normalized by the cell number of each experimental group. α-tubulin was an internal control for Western blot. U6 was an internal control for qRT-PCR. A *p*<0.05 was considered statistically significant.

## Results

### Down-regulation of miR-34a and miR-125b in a human diabetic heart

To evaluate the roles of miRNAs in a human diabetic heart, miR-34a and miR-125b expression levels were examined in human heart tissues obtained from 20 patients with diabetic heart failure and 20 healthy donors. The expressions of miR- 34a and miR-125b were significantly downregulated in heart tissues with diabetic heart failure, when compared with those from healthy donors (Figure 1A and 1B). These results suggest a protective role of miR-34a and miR-125b during high glucose-mediated heart failure.

**Figure 1:** Down-regulation of miR-34a and miR-125b in a human diabetic heart, induced by hyperglycemia. (A) Expressions of miR-34a and (B) miR-125b in a normal human heart and diabetic heart tissues. (C) Rat cardiomyocytes were treated with control or high glucose at 5, 10, or 20 mM. The relative expressions of miR-34a and (D) miR-125b were assessed by real-time PCR and normalized to U6 snRNA levels. Columns, mean of three independent experiments; the error bars in graphs represented SD. **, p < 0.01; ***, p < 0.001.

### Short-term high glucose stimulates miR-34a and miR-125b expressions in neonatal rat cardiomyocytes

To evaluate the effect of miR-34a and miR-125b in high glucose-induced cardiomyocyte dysfunction, this study established an *in vitro* model using the isolated rat cardiomyocytes cultured under normal and high glucose conditions. Interestingly enough, within a short period of time (1 hour), miR-34a and miR-125b were found to be significantly induced by 25 or 50 mM high glucose concentration (Figure 1C and 1D). However, under long time hyperglycemia (48 hours, 50 mM), cardiomyocytes underwent cell death (data not shown). Taken together, the above results revealed an adaptive upregulation of miR-34a and miR-125b by hyperglycemia in cardiomyocytes.

### Overexpression of miR-34a and miR-125b protects against hyperglycemia-induced cardiomyocyte death

It is well-known that hyperglycemia can trigger inflammatory responses and induce cardiomyocyte cell death (4). It was therefore asked whether exogenous overexpressing miR-34a and miR-125b could protect against hyperglycemia-induced cardiomyocyte death. Rat cardiomyocytes were co-transfected with pre-miR-34a and pre-miR-125b for 48 hours. qRT-PCR results showed miR-34a and miR-125b expressions were increased by 5-10 folds (Figure 2A). Cells were then exposed to 25 or 50 mM glucose to mimic hyperglycemia for 48 hours. Expectedly, control miRNA transfected cardiomyocytes presented a clear cell death under HG treatment (Figure 2B). However, cardiomyocytes with miR-34a and miR-125b overexpression proved to be significantly resistant to HG when analyzed by both MTT assay and Caspase-3 activity assay (Figure 2B and 2C). These results demonstrated a protective role of miR-34a and miR-125b in HG-induced cardiomyocyte cell death.

**Figure 2:** Overexpression of miR-34a and miR-125b protects against hyperglycemia-induced cardiomyocytes death. (A) Rat primary cardiomyocytes were transfected with control microRNA, or pre-miR-34a plus miR-125b for 72 hours. The expressions of miR-34a and miR-125b were measured by qRT-PCR. (B) Rat primary cardiomyocytes were transfected with control microRNA, or pre-miR-34a plus miR-125b for 72 hours; cells were exposed under normal or high glucose (25 mM or 50 mM) for 48 hours. The cell apoptosis rate was measured by MTT assay and (C) Caspase-3 activity. Columns, mean of three independent experiments; the error bars in graphs represented SD. *, p < 0.05; **, p < 0.01; ***, p < 0.001.

### Inhibition of glycolysis by miR-34a and miR-125b under hyperglycemia by targeting LDHA and HK2

Under high glucose, cells exhibited an increased cellular glucose metabolism.^6^^,^^7^ To investigate the mechanisms underlining the roles of miR-34a and miR-125b during HG treatments, a literature review was performed and recent studies were found that showed both miR-34a and miR-125b could inhibit intracellular glucose metabolism.^15^^,^^16^ Thus, it was hypothesized that the inhibition of the HG-activated intracellular glycolysis by miR-34a and miR-125b contributes to protecting cardiomyocytes against cell death. To assess the inhibitory effects of miR-34a and miR-125b on glycolysis under hyperglycemia, the glucose uptake and lactate production of cardiomyocytes with or without miRNAs overexpression were measured in normal or high glucose treatments. Overexpression of miR-34a and miR-125b inhibited glucose uptake and lactate production under normal glucose (Figure 3A and 3B). In addition, cardiomyocytes with high miR-34a and miR-125b also displayed significantly decreased glycolysis, close to the normal levels (Figure 3A and 3B), suggesting that an overexpression of miR-34a and miR-125b under hyperglycemia contributes to the maintenance of intracellular glucose homeostasis. To verify the direct targets of miR-34a and miR-125b in cardiomyocytes, we detected the protein expressions of LDHA, which is a predicted target of miR-34a and HK2, the 3’UTR of which could be directly targeted by miR-125b (Figure 4A). Western blot results consistently showed the protein levels of HK2 and LDHA were suppressed by an overexpression of miR-34a and miR-125b under both normal and HG conditions (Figure 4B). The targets of miR-34a and miR-125b were further verified in human heart tissues. In a consistent manner, in miR-34a and miR- 125b, whose expression is relatively high in normal heart tissues, the mRNA levels of HK2 and LDHA were apparently low (Figure 4C). The same negative correlation between miR-34a and LDHA, miR-125b, and HK2 was observed in diabetic heart tissues (Figure 4D). In general, our data support that the miR-34a and miR-125b inhibit intracellular glycolysis by targeting glycolysis speed limited enzymes, contributing to the maintenance of glucose homeostasis under hyperglycemia.

**Figure 3:** Overexpression of miR-34a and miR-125b impairs hyperglycemia-induced glucose metabolism. (A) Rat primary cardiomyocytes were transfected with control microRNA, or pre-miR-34a plus miR-125b for 72 hours; cells were exposed under normal or high glucose (25 mM) for 48 hours. Glucose uptake and (B) lactate production were measured. Columns, mean of three independent experiments; the error bars in graphs represented SD. *, p < 0.05; **, p < 0.01; ***, p < 0.001.

**Figure 4:** miR-34a and miR-125b target glycolysis enzymes in cardiomyocytes and heart tissues. (A) illustration of LDHA 3’UTR and HK2 3’UTR, as well as the seed sequence of miR-34a and miR-125b, showing the computationally predicted target region on the 3’UTR of LDHA and HK2 mRNAs. (B) Rat cardiomyocytes were transfected with 25 nM miR-34a plus miR-125b precursors for 72 h. Overexpression of miR-34a plus miR-125b downregulated LDHA and HK2 protein expressions under normal and high glucose conditions. α-Tubulin was a loading control. (C) Negative correlation between miR-34a and LDHA mRNA expressions in normal human heart tissues. (D) Negative correlation between miR-125b and HK2 mRNA expressions in normal human heart tissues.

### Restoration of LDHA and HK2 sensitizes cardiomyocyte to high glucose

Finally, to test whether the protection of cardiomyocytes under HG was directly caused by glycolysis inhibition by miR-34a and miR-125b, rescue experiments were performed by co-transfection of LDHA and HK2 overexpression plasmids into miR-34a and miR-125b overexpressing cardiomyocytes. Western blot results (Figure 5A) showed that the co-transfection of plasmids successfully rescued the LDHA and HK2 expressions in miR-34a and miR-125b overexpressing cardiomyocytes. Furthermore, the glucose uptake (Figure 5B) and lactate production (Figure 5C) were also recovered to normal levels by restoration of LDHA and HK2. The above transfected cells were exposed to 50 mM glucose to mimic hyperglycemia for 48 hours. Cells were collected and subjected to cell death assay. As expected, cardiomyocytes with LDHA and HK2 rescue showed significantly increased cell death under hyperglycemia, as compared to that of miR-34a and miR-125b overexpressing cardiomyocytes (Figure 5D). These rescue experiments supported the miR-34a and miR-125b-mediated intracellular glucose homeostasis in hyperglycemia directly protected by cardiomyocytes.

**Figure 5:** Restoration of glycolysis in miR-34a and miR-125b overexpressed rat cardiomyocyte promotes cell death under hyperglycemia. (A) Rat cardiomyocytes were transfected with miR-34a plus miR-125b mixture alone or co-transfected with miR-34a plus miR-125b and LDHA plus HK2 overexpression plasmids for 72 hours. Cells were then treated with or without high glucose (25 mM) for 48 hours and subjected to Western blot analysis. α-Tubulin was a loading control. (B) Rat cardiomyocytes were transfected with miR-34a plus miR-125b mixture alone or co-transfected with miR-34a plus miR-125b and LDHA plus HK2 overexpression plasmids for 72 hours. Cells were then treated with or without high glucose (25 mM) for 48 hours. Glucose uptake and (C) lactate production were measured. (D) Rat cardiomyocytes were transfected with miR-34a plus miR-125b mixture alone or co-transfected with miR-34a plus miR-125b and LDHA plus HK2 overexpression plasmids for 72 hours. Cells were then treated with or without high glucose (50 mM) for 48 hours. Cell death was assessed by MTT assay. Columns, mean of three independent experiments; the error bars in graphs represented SD. *, p < 0.05; **, p < 0.01; ***, p < 0.001.

## Discussion

DCM is one of the major health threats in patients with diabetes.^1^^,^^2^ It is associated with complex pathophysiologic events, including chronic inflammation and cardiac cell death, eventually resulting in heart failure. The early cardiac response to diabetes was apoptotic cardiomyocyte death.^3^ The present study reported on a microRNA-mediated cardiomyocyte protection mechanism under hyperglycemia. Rat cardiomyocytes were treated with high glucose, identifying a significant increased glycolysis, a process regulated by the adaptively upregulated miR-34a and miR-125b, indicating that the targeting of hyperglycemia-induced glucose metabolism by miR-34a and miR-125b might contribute to the development of a therapeutic method to protect against cardiac cell death.

Glucose and acute insulin resistance have been found in acute cardiac conditions.^4^ Furthermore, high intracellular glucose metabolism has been recognized as a potential prognostic marker in acute coronary syndromes.^17^ The association between hyperglycemia and DCM has been extensively studied.^18^ Abnormal insulin metabolic signaling, hyperglycemia, mitochondrial dysfunction, and oxidative stress are the most recognized pathophysiological mechanisms involved in the development of DCM.^18^ Currently, the underlying molecular mechanisms resulting in DCM are poorly understood. Recent studies have shown that microRNAs play essential roles in the etiology of diabetes and its complications.^11^ Moreover, miR-125b and miR-34a have proven to be associated with the oxidative stress of cardiac tissues, leading to prevent against cardiomyocytes cell death.^11^ In cancers, miR-34a and miR-125b have been reported to downregulate and inhibit glucose metabolism,^15^^,^^19^ suggesting that miR-34a and miR-125b could regulate the dysfunctional cells under hyperglycemic conditions. Our results demonstrated that miR-34a and miR-125b were significantly downregulated in human diabetic heart tissues, when compared with a normal heart. It was also found that miR-34a and miR-125b were adaptively stimulated under high glucose conditions in rat cardiomyocytes, suggesting that miR-34a and miR-125b might suppress the hyperglycemia-induced intracellular glucose metabolism. It is well-known that miR-34a could target 3’UTR of LDHA mRNA^20^ and that HK2 is a direct target of miR-125b in cancer cells.^19^ This study has some limitations in confirming the effects of the miRNAs on the DCM protection from *in vivo* data. Our data, however, has, for the first time, revealed that miR-34a and HK2 could suppress glucose uptake and lactate production in cardiomyocytes. Overexpression of miR-34a and miR-125b contributed to the maintenance of intracellular glucose metabolism under hyperglycemic conditions (Figure 3).

## Conclusion

In summary, the present study demonstrated that miR-34a and miR-125b were significantly correlated with human DCM. Using an *in vitro* rat cardiomyocytes model, hyperglycemia adaptively stimulated miR-34a and miR-125b expressions. The overexpression of miR-34a and miR-125b suppressed high glucose-induced intracellular glucose metabolism by targeting LDHA and HK2, resulting in the prevention of the hyperglycemia-induced cardiomyocyte cell death. Taken together, this study serves to reveal the potential roles of miR-34a and miR-125b in the pathogenesis of hyperglycemia-induced cardiomyopathy. Our future work will focus on an *in vivo* rat diabetic model to investigate the molecular mechanisms of miR-34a and miR-125b in DCM.
